# National trends of wrist arthroscopy in Italy: Analysis from 2001 to 2016

**DOI:** 10.1002/jeo2.70193

**Published:** 2025-03-07

**Authors:** Umile Giuseppe Longo, Rocco Papalia, Alessandro Mazzola, Sergio De Salvatore, Alessandro Tancioni, Valentina Piccioni, Alessandro de Sire, Kristian Samuelsson, Stefano Zaffagnini, Ilaria Piergentili, Pieter D'Hooghe, Vincenzo Denaro

**Affiliations:** ^1^ Fondazione Policlinico Universitario Campus Bio‐Medico Roma Italy; ^2^ Research Unit of Orthopaedic and Trauma Surgery, Department of Medicine and Surgery Università Campus Bio‐Medico di Roma Roma Italy; ^3^ IRCCS Ospedale Pediatrico Bambino Gesù Rome Italy; ^4^ Department of Medical and Surgical Sciences University of Catanzaro “Magna Grecia” Catanzaro Italy; ^5^ Department of Orthopaedics, Institute of Clinical Sciences, The Sahlgrenska Academy University of Gothenburg Gothenburg Sweden; ^6^ Clinica Ortopedica e Traumatologica II IRCCS Istituto Ortopedico Rizzoli Bologna Italy; ^7^ CNR‐IASI, Laboratorio di Biomatematica, Consiglio Nazionale delle Ricerche Istituto di Analisi dei Sistemi ed Informatica Rome Italy; ^8^ Department of Orthopaedic Surgery and Sportsmedicine Aspetar Hospital Doha Qatar

**Keywords:** analysis, arthroscopy, economy, epidemiology, Italy, surgery, wrist

## Abstract

**Purpose:**

This study aimed to evaluate the demographic features of patients undergoing wrist arthroscopy in Italy. A secondary aim was to perform an economic analysis of this type of surgery.

**Methods:**

The National Hospital Discharge Records database was employed to conduct the analysis. Wrist arthroscopy surgical procedures were defined by the primary procedure code 80.23, according to the International Classification of Diseases, Ninth Revision–Clinical Modification code. Incidence rates were computed by dividing the number of annual cases by the size of the adult population reported annually by the National Institute for Statistics.

**Results:**

7875 wrist arthroscopy procedures were performed in Italy. The cumulative incidence rate was 1 for every 100,000 Italian residents. The need for wrist arthroscopy in Italy increased from 2001 to 2008 and then progressively declined up to 2016. The highest number of procedures was found between 40 and 49 years. Most patients undergoing wrist arthroscopy were females (50.6%). The mean age of patients was 41.6 ± 14.5. Wrist arthroscopy in Italy costs an average of 721,102 ± 171,195€ each year.

**Conclusions:**

The incidence of this type of surgery peaked throughout the course of the 15 years, in 2006 and 2008. However, the number of procedures per 100,000 inhabitants has decreased since 2008. The economic analysis revealed that the cost of wrist arthroscopy is relevant to the healthcare system in Italy.

**Level of Evidence:**

Level II.

**Abbreviations**
CT: computed tomography
DRG: diagnosis‐related group
ICD‐9‐CM: International Classification of Diseases, Ninth Revision–Clinical Modification code
ISTAT: National Institute for Statistics
MRA: magnetic resonance arthrogram
MRI: magnetic resonance imaging
M/F: male/female
NHDR: National Hospital Discharge Records
NHS: National Health Service
PLD: perilunate dislocation
PLFD: perilunate fracture dislocation
TFCC: triangular fibrocartilage complex

## INTRODUCTION

Chen described the first wrist arthroscopy in 1979 [6]. Since its first use more than 40 years ago, the indications for wrist arthroscopy have increased along with technological advancements and anatomical understanding [39]. This approach reduces exposures and provides minimally invasive access to anatomical regions that would otherwise be hardly accessible: it enables a direct, magnified and tactilely assisted inspection [39]. Despite the cost and complexity of arthroscopy procedures, wrist arthroscopy is considered the most sensitive and specific option for evaluating carpal abnormalities: it allows direct visualization of injuries, enables functional testing and offers a minimally invasive treatment at the same time [12, 38]. Kader et al. [14] showed that, when compared to the gold standard of wrist arthroscopy, neither magnetic resonance imaging (MRI) nor MR arthrogram (MRA) scanning is sensitive enough in the diagnosis of scapholunate interosseous ligament injury. Although Omar et al. [28] demonstrated that, for both central and ulnar triangular fibrocartilage complex (TFCC) injuries, MRA and arthroscopy have a nearly complete agreement, the literature on the diagnostic accuracy of MRI and MRA varies greatly [5, 11, 29, 33, 34, 35].

In a systematic review by Liechti et al. [20], surgically treated perilunate dislocations (PLDs) and perilunate fracture dislocations (PLFDs) were analyzed, showing much higher complication rates with open surgery versus arthroscopy (17.4% vs. 4.8%). Accordingly, the authors suggested that to reduce post‐operative complications, arthroscopic surgery should be performed whenever feasible [20].

Clark et al. [7] analyzed outcomes of open versus arthroscopic ganglion cyst excision, showing the advantages of the arthroscopic treatment in terms of both traditional surgical results and patient‐centred outcomes.

According to the literature, wrist arthroscopy complications are rare, occurring at a rate of about 2% [9, 10, 22]. The majority of them arise from injuries caused by arthroscopic instrumentation in a restricted joint space. Due to reported lesions to the extensor tendons, radial and ulnar artery and nerves, caution must be exercised when creating portals [1, 9, 10]. Additionally, infections and reflex sympathetic dystrophy have been documented [1, 9].

In general, when compared to typical open procedures, wrist arthroscopy provides a more detailed understanding of the anatomy and mechanisms of disease and frequently results in a more precise diagnosis [31]. In addition, arthroscopic treatment has fewer surgical complications, less post‐operative discomfort, less surgical tissue stress, and shorter recovery times [31].

To our knowledge, national registers regarding wrist arthroscopy have not been published by the majority of the developed countries. The epidemiology of patients undergoing wrist arthroscopy in the Italian population has not been sufficiently studied. Understanding the demographic trends of patients requiring wrist arthroscopy procedures may be significant for better addressing the future of this surgery and the related health service planning. One of the basic principles of the Italian National Health Service (NHS) is equity in access to healthcare; patients in Italy are given free access to the NHS. This study aimed to estimate the annual number of wrist arthroscopy procedures in Italy from 2001 to 2016 and the patients' demographic characteristics. A secondary aim was to perform an economic analysis of this type of surgery in Italy.

## MATERIALS AND METHODS

This report is a nationwide registry study of all wrist arthroscopy surgical procedures performed between 2001 and 2016 in all public and private hospitals in Italy. The Italian Ministry of Health's official diagnosis and procedure database, the National Hospital Discharge Records (NHDR), was used to collect patient data. The features of patients (age and gender), hospitalization stay length, diagnosis, and procedure are all part of this database. Wrist arthroscopy surgical procedures were defined by the primary procedure code 80.23, according to the International Classification of Diseases, Ninth Revision–Clinical Modification code (ICD‐9‐CM). The National Institute for Statistics (ISTAT) adult population data for each year were also utilized to determine the incidence rates. The incidence rates were stratified by gender, age group and year. This study referred to the adult population (i.e., ≥15 years of age). The present study is based on official data sources, such as hospitalization records. The data sets used and/or analyzed during the current study are available from the corresponding author on reasonable request. The Institutional Review Board of Campus Bio‐Medico University of Rome ruled that no formal ethics approval was required in this particular case. All data were obtained by the Direzione Generale della Programmazione Sanitaria—Banca Dati SDO of the Italian Ministry of Health.

All patients included in the present study were codified with a primary procedure code 80.23, according to the ICD‐9‐CM, standing for wrist arthroscopy. The exclusion was applied when a diagnosis code associated with that wrist arthroscopy was atypical and did not apply to the 80.23 code.

### Statistics

The R program, a statistical computer and graphical software environment, was used to conduct a number of descriptive statistical analyses. Microsoft Excel (2019) and IBM SPSS Statistics for Windows, version 26.0. (Armonk, NY, USA: IBM Corp.) were used. Descriptive statistics were performed (mean and standard deviation for continuous data, frequencies and percentages for categorical data). The annual number of surgical procedures divided by the annual size of the adult population (≥15 years old) per 100,000 residents was calculated to define the incidence rates of wrist arthroscopy.

According to the Ministerial Decree (18 December 2008), analyses of estimated costs were based on the cost attributed to diagnosis‐related groups (DRGs). Regardless of the diagnosis, the procedure's complexity, or the patient's health at admission, all DRG procedures in Italy are reimbursed equally. The average of the lowest and greatest possible reimbursement value has been used to compute economic reimbursement for each individual year. These excursion ranges are explained by the fact that reimbursement in Italy does differ by area.

## RESULTS

### Demographics

**Figure 1:**
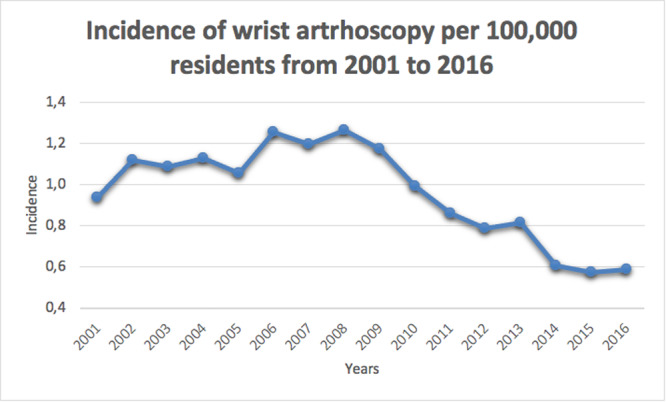
Incidence of surgical procedures for wrist arthroscopy (≥15 years of age) per 100,000 residents from 2001 to 2016 in Italy.

**Figure 2:**
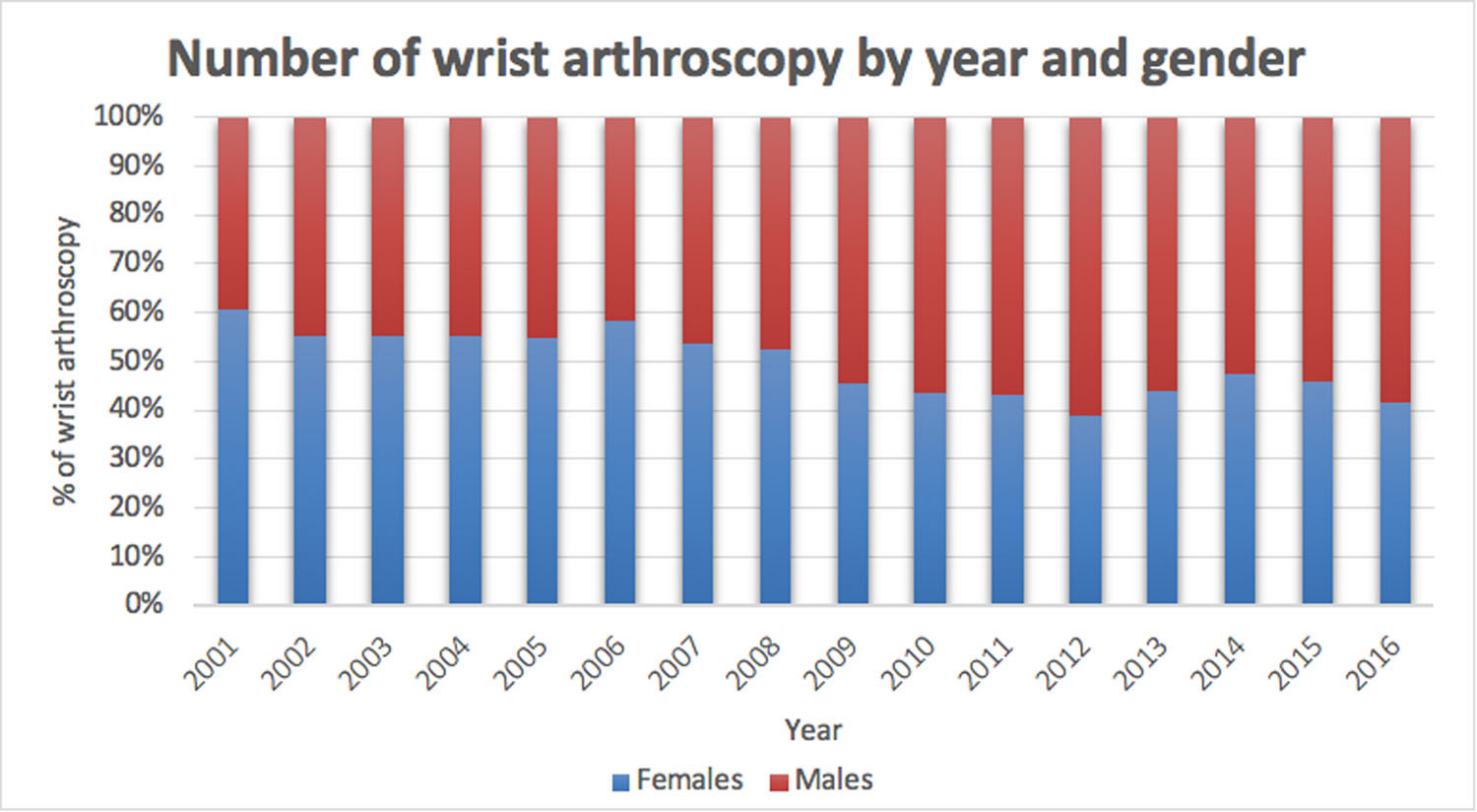
Number of surgical procedures for wrist arthroscopy performed in Italy from 2001 to 2016, stratified by gender.

**Figure 3:**
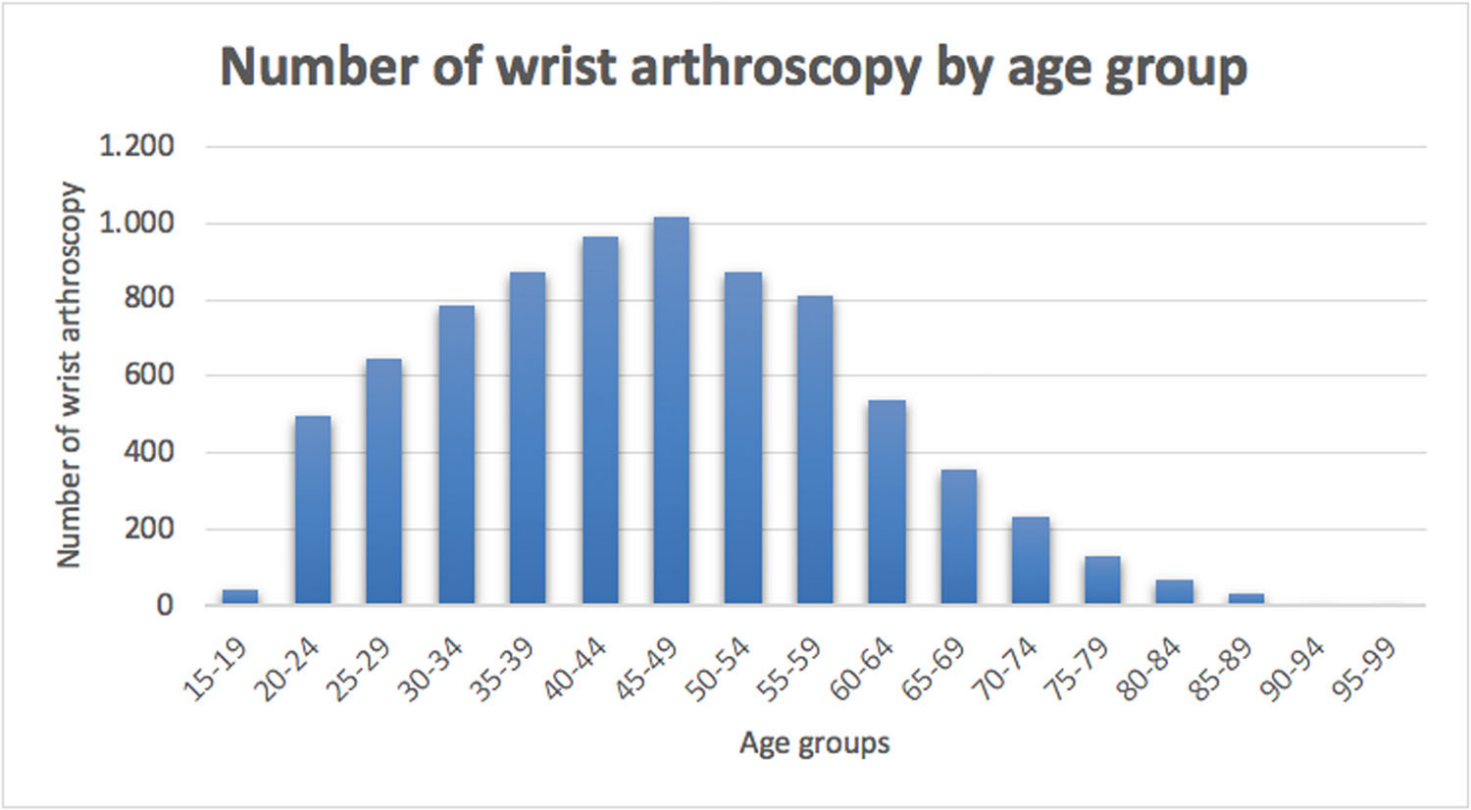
Number of surgical procedures for wrist arthroscopy performed in Italy from 2001 to 2016, stratified by age groups.

**Figure 4:**
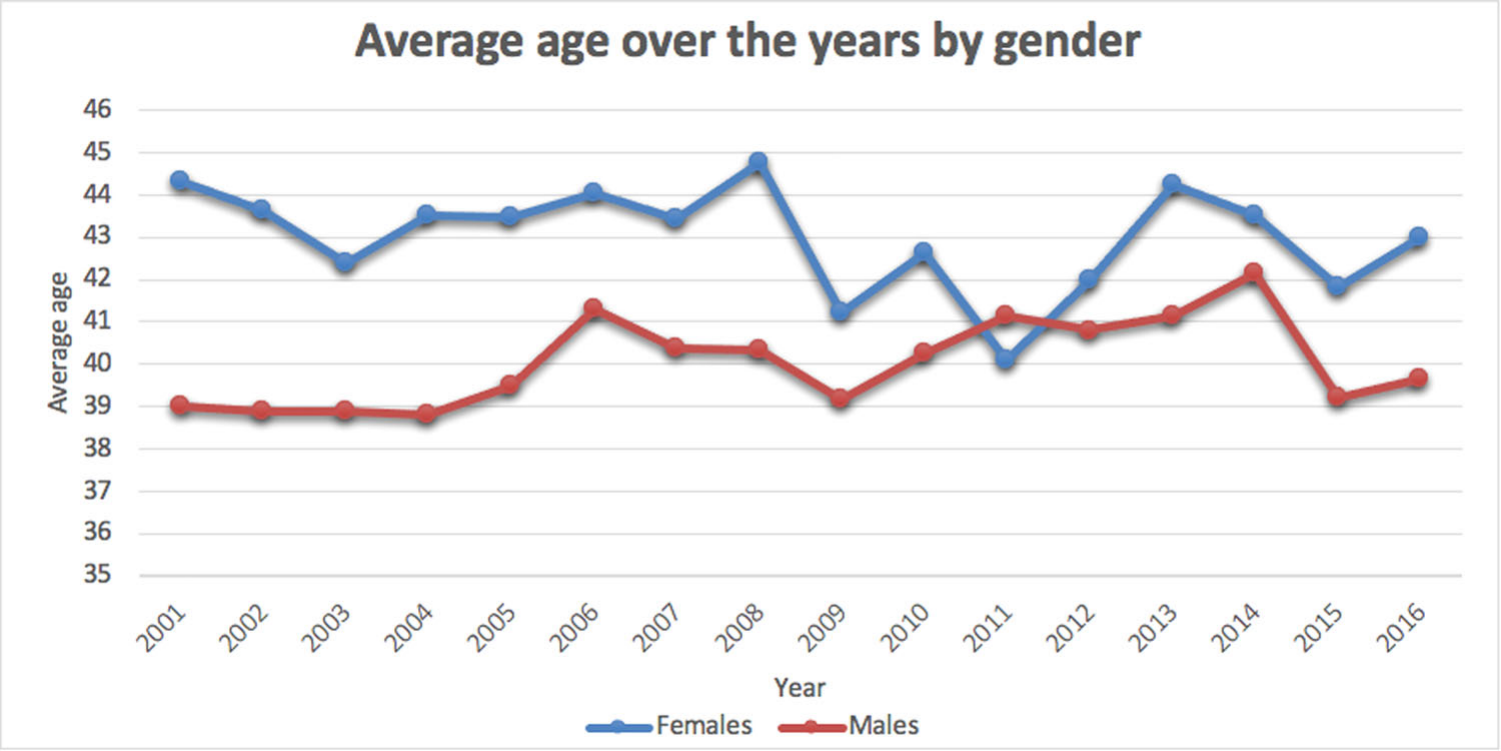
Average age of patients requiring wrist arthroscopy over the years by gender.

In the 16‐year study period, 7875 wrist arthroscopy procedures were performed in Italy. The cumulative incidence rate was 1 for every 100,000 Italian residents between 2001 (0.9 cases per 100,000 residents) and 2016 (0.6 cases per 100,000 residents). The peak was 1.3 in 2006 and 2008 (Figure 1). The male/female (M/F) ratio varied from a minimum of 0.7 (2001) to a maximum of 1.4 (2016), with an overall average M/F ratio of 1. Most patients undergoing wrist arthroscopy were females (50.6% of females and 49.4% of males). In addition, females represented the majority of patients between 2001 and 2008 (Figure 2). During the 16‐year study period, the highest number of wrist arthroscopy procedures was found between 40 and 49 years (Figure 3). From 2001 to 2016, the mean age of patients was 41.6 ± 14.5 (43.2 ± 15 years for females and 40 ± 13.7 years for males). Overall, the average age of female patients was constantly higher than that of male patients, except in 2011 (Figure 4).

### Length of the hospitalization

**Figure 5:**
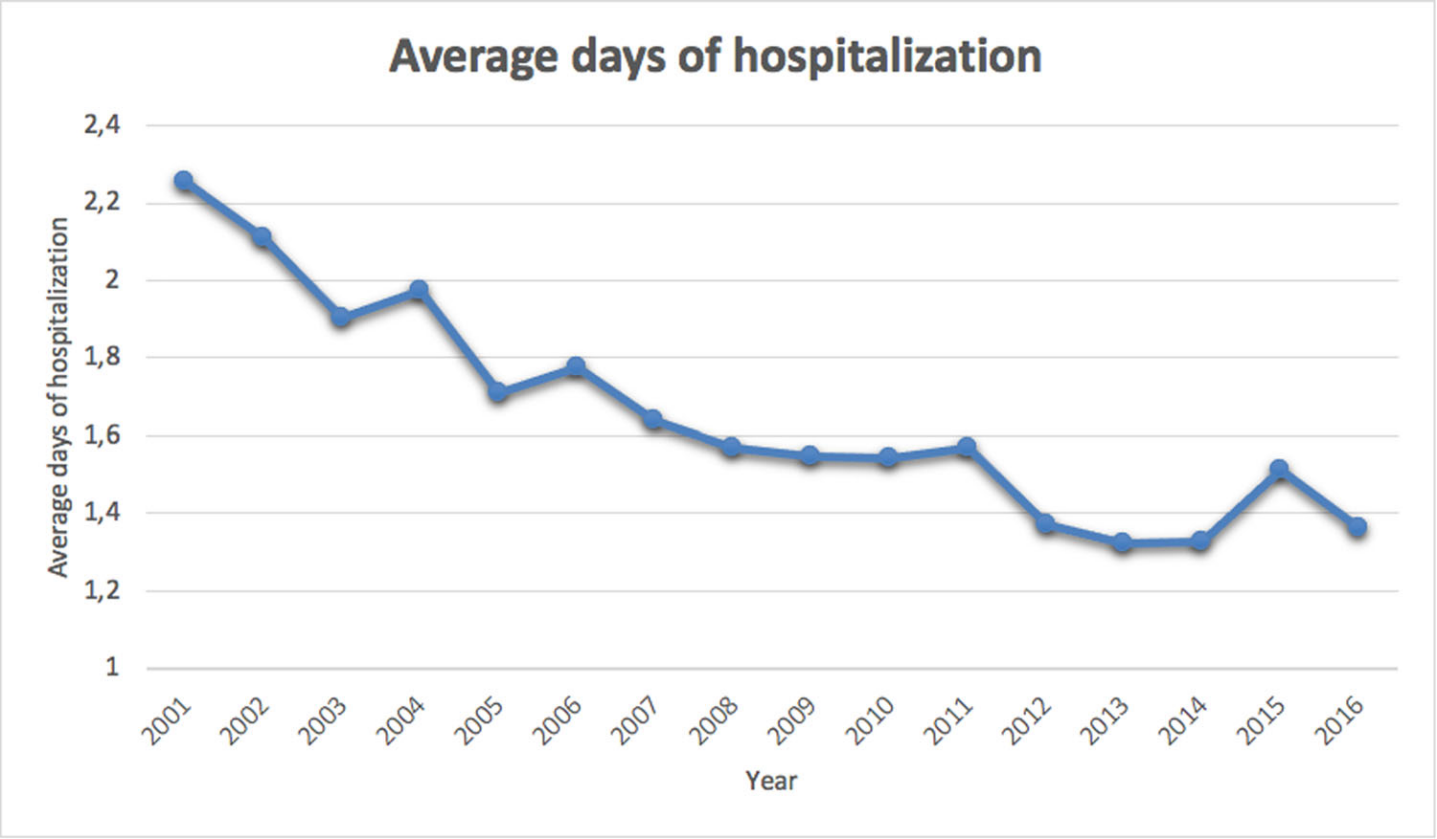
Average days of hospitalization in the study period.

**Figure 6:**
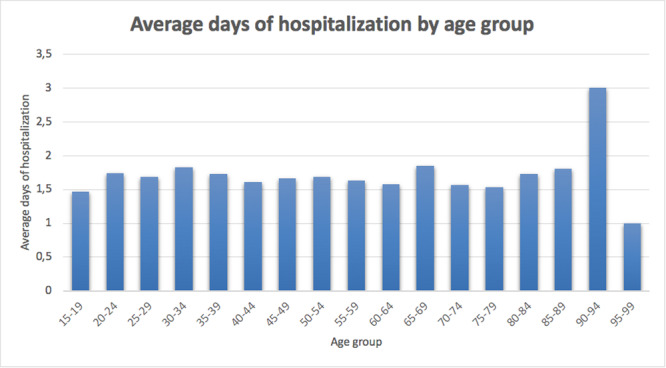
Average days of hospitalization by age groups in the study period.

The average length of hospital stay was 1.7 ± 1.7 days (ranging from 1 to 58 days), with a decreasing trend (Figure 5). Males required, on average, 1.7 ± 1.9 days of hospital stay, whereas females 1.6 ± 1.4 days. On average, patients aged from 90 to 94 required more days of hospital stay (Figure 6).

### Main primary diagnoses

During the 16 years, the main primary diagnoses were Carpal Tunnel Syndrome (ICD code: 354.0; 10.7%), Distortion and Radiocarpal Distraction (Articulation) (Ligament) (ICD code: 842.02; 8.2%) and Fracture After‐Effect of the Upper Limbs (ICD code: 905.2; 6.8%).

### Economic impact

To date, for each hospital admission for wrist arthroscopy, the average Italian hospital reimbursement varies from 1361€ (1‐day stay treatment) to 1512€ (more than 1‐day stay, with an increase of 7€ for each additional day of hospital stay). Between 2001 and 2016, a total cost of 11,537,641€ was calculated. Wrist arthroscopy surgical procedures in Italy cost an average of 721,102 ± 171,195€ each year, with costs ranging from 439,880€ in 2015 to 963,044€ in 2008. These excursion ranges can be explained by the fact that reimbursement differs from region to region in Italy.

## DISCUSSION

The incidence of wrist arthroscopy and its trends in the Italian population are the study's most significant findings. This study is the first to give population‐based national rates for arthroscopic procedures on the wrist in Italy. The number of procedures carried out in Italian public and private hospitals over the course of 15 years was calculated using a validated national registry. In the current literature, there is a lack of epidemiological studies on this topic. The use of wrist arthroscopy as a diagnostic tool and as part of therapeutic interventions is becoming popular. Accordingly, the number of studies on wrist arthroscopy that are published each year is rising [27]. Currently, various wrist diseases can be evaluated arthroscopically for diagnostic purposes: osteochondral lesions of the carpus, injuries to the triangular TFCC, wrist pain of unknown origin, synovial biopsy and dynamic evaluation of carpal instability and radiocarpal arthritis [1, 39]. Emerging therapeutic uses now include arthroscopy‐assisted fracture reductions, radiocarpal synovitis and arthritis treatment, bacterial sampling and joint lavage [1, 39]. Moreover, promising is the arthroscopic approach to soft tissue wrist diseases such as ganglion excisions, TFCC repairs, lunotriquetral instability, PLD, scapholunate instability, scapholunate ligament reconstruction, distal radioulnar joint stabilization and contracture release, carpal tunnel syndrome [1, 16, 21, 24, 39]. Four randomized trials that compared the arthroscopic wrist treatment with an open procedure were identified in a systematic review that was conducted by Tadjerbashi et al. [37]. Many other studies in the literature advocate arthroscopic wrist procedures as a rational substitute or assistance for open surgical approaches since they produced better cosmetic results and decreased post‐operative morbidity in the treatment of Kienböek disease [19], in the excision of dorsal and volar ganglion [15, 32], in the management of distal radius [26] and scaphoid fractures [8].

In Italy, the incidence of this type of surgery peaked over the course of 15 years, in 2006 and 2008. However, the number of procedures per 100,000 people has decreased since 2008. Potential explanations of this behaviour could be the start of the economic crisis that might have played a role, as well as the improvement of noninvasive imaging techniques (3‐T MRI). However, no data can confirm this claim. Data of the present study are in line with a Finnish nationwide analysis reporting an increasing incidence rate of wrist arthroscopy on average by 15% every year until 2014, followed by a decline on average by 4% every year [17]. The fact that MRI and computed tomography (CT) scans are used more frequently in recent years as diagnostic techniques in place of arthroscopy can justify these decreasing trends, limiting the role of wrist arthroscopy as a diagnostic tool for joint exploration [27]. Interestingly, the same Finnish study showed a reduction in all arthroscopic procedures [17]. Recent research demonstrating the lack of efficacy of various common knee and shoulder arthroscopic procedures can explain the declining frequencies of these procedures and the rising need for conservative treatment [2, 3, 18, 23, 36]. However, the reasons why the frequency of elbow, hip and wrist arthroscopy procedures (as confirmed by the present study) is likewise declining have not yet been documented in clinical trial studies. It can be assumed that economic analyses and the rising demand for the cost‐effectiveness of healthcare interventions are among the factors that might have had an impact on the rate of arthroscopy procedures. Between the ages of 40 and 49, the incidence of wrist arthroscopy peaked in Italy. Similar results from the literature confirmed that it is mainly a young patients' surgery, with the majority of patients aged 15–44 years [13, 17, 40].

A hospital‐based study in China found that nearly two‐thirds (62%) of the patients treated were males [40]. The present study showed an overall average M/F ratio of 1 in the Italian population. Interestingly, Billig et al. [4] found that wrist arthroscopy patients receive different preoperative care depending on their gender. While women employ more conservative measures, suggesting potential gender disparities in illness presentation and potential implicit provider biases in preoperative therapy, males utilize more imaging, suggesting a more thorough preoperative evaluation for wrist discomfort [4].

Over the study period, the length of hospitalization progressively decreased, with men reporting a slightly longer length of hospital stay. The variation is most likely the effect of hospitals generally reducing the length of stay over time for financial reasons. However, no data can confirm this claim. The analysis of the primary diagnosis codes revealed that carpal tunnel syndrome, ligament sprains and wrist fractures are the leading indications for wrist arthroscopy in Italy. These results are similar to data from other countries, underlying the potential role of minimally invasive surgery in wrist disorders worldwide [13, 17, 40]. Yin et al [40], found that in China, the largest group of patients who underwent wrist arthroscopy are those who complained of ulnar‐sided wrist pain; unfortunately, in the present study, the standardized structure of the ICD‐9‐CM did not allow to specify the exact origin of wrist pain.

Ponkilainen et al. [30], in a study on the Finnish population, found substantial regional disparities in the spread of wrist arthroscopy: incidence rates were higher in hospitals with smaller populations and higher historical incidence rates, indicating that small non‐academic hospitals are slower to adopt evidence. Further studies are needed to investigate whether similar regional differences may be observed in Italy. The economic burden of wrist arthroscopy in Italy was reported in the results of the present study: depending on the length of stay, the admission reimbursement in Italy ranges from 1361 to 1512€, with an average cost of 721,102 ± 171,195€ each year. The analysis conducted by Malik et al. in the same period of the present study showed that Medicare reimbursement for hand surgery operations, including wrist arthroscopy, has decreased over time [25]. Health policymakers need to monitor the effects of declining reimbursements when updating national reimbursement standards in order to both guarantee provider satisfaction and maintain patient access to care.

This research has several limitations. First, the ICD‐9‐CM used in this study is based on administrative data from different hospitals and regions. It serves as the starting point for all reported diagnoses and procedures, but these data are prone to errors. Because there are so many hospitals involved, it is challenging to recognize incorrect diagnoses or coding errors. Second, this study lacks outcome scores; in Italy's healthcare system, hospitalizations are anonymous, therefore patients do not receive a unique ID number. In simple terms, patients who underwent multiple procedures may have been counted more than once. Third, since surgeons performed the ICD‐9 classification, there may be variations between observers.

## CONCLUSIONS

Overall, 7875 wrist arthroscopy procedures were performed in Italy from 2001 to 2016. The incidence of this type of surgery peaked throughout the course of the 15 years, in 2006 and 2008. However, the number of procedures has progressively decreased since 2008. The age groups from 40 to 49 years showed the highest need for wrist arthroscopy. The economic analysis revealed that the cost of wrist arthroscopy is relevant to the healthcare system in Italy. Analysis of demographic trends and improved understanding of charge and reimbursement patterns of patients requiring wrist arthroscopy provides policymakers with an opportunity to enhance resource management across the orthopaedics field.

## AUTHOR CONTRIBUTIONS

**Umile Giuseppe Longo**: Conceptualization; methodology; formal analysis; investigation; data curation; supervision; project administration; writing—review and editing. **Rocco Papalia**: Conceptualization; formal analysis; writing—review and editing. **Alessandro Mazzola**: Methodology; formal analysis; writing—original draft preparation. **Sergio De Salvatore**: Methodology; supervision. **Alessandro Tancioni**: Methodology; writing—original draft preparation. **Valentina Piccioni**: Methodology; writing—original draft preparation. **Ilaria Piergentili**: Software; validation; formal analysis. **Kristian Samuelsson**: Software; validation; formal analysis; writing—review and editing. **Alessandro de Sire**: Investigation; visualization. **Pieter D'Hooghe**: Investigation; visualization. **Stefano Zaffagnini**: Data curation. **Vincenzo Denaro**: Writing—review and editing; project administration. All authors have read and agreed to the published version of the manuscript.

## CONFLICT OF INTEREST STATEMENT

The authors declare no conflicts of interest.

## ETHICS STATEMENT

The Institutional Review Board of Campus Bio‐Medico University of Rome ruled that no formal ethics approval was required in this particular case, and the need to obtain informed consent was waived based on the retrospective design and anonymization of patient identifiers (Prot. number: 113/20 (OSS) ComEt UCBM). All methods were performed in accordance with the relevant guidelines and regulations.

## Data Availability

The data sets used and/or analyzed during the current study are available from the corresponding author on reasonable request. Access to the database is also on request. All data were obtained by the Direzione Generale della Programmazione Sanitaria—Banca Dati SDO of the Italian Ministry of Health.
